# Early neural disruption and auditory processing outcomes in rodent models: implications for developmental language disability

**DOI:** 10.3389/fnsys.2013.00058

**Published:** 2013-10-21

**Authors:** R. Holly Fitch, Michelle L. Alexander, Steven W. Threlkeld

**Affiliations:** ^1^Department of Psychology/Behavioral Neuroscience, University of ConnecticutStorrs, CT, USA; ^2^Department of Pediatrics, University of MinnesotaMinneapolis, MN, USA; ^3^Department of Psychology, Rhode Island College, ProvidenceRI, USA

**Keywords:** rapid auditory processing, language disability, cortical lesion, timing effects, plasticity and injury, rodent models, medial geniculate nucleus

## Abstract

Most researchers in the field of neural plasticity are familiar with the “Kennard Principle,” which purports a positive relationship between age at brain injury and severity of subsequent deficits (plateauing in adulthood). As an example, a child with left hemispherectomy can recover seemingly normal language, while an adult with focal injury to sub-regions of left temporal and/or frontal cortex can suffer dramatic and permanent language loss. Here we present data regarding the impact of early brain injury in rat models as a function of type and timing, measuring long-term behavioral outcomes via auditory discrimination tasks varying in temporal demand. These tasks were created to model (in rodents) aspects of human sensory processing that may correlate—both developmentally and functionally—with typical and atypical language. We found that bilateral focal lesions to the cortical plate in rats during active neuronal migration led to worse auditory outcomes than comparable lesions induced after cortical migration was complete. Conversely, unilateral hypoxic-ischemic (HI) injuries (similar to those seen in premature infants and term infants with birth complications) led to permanent auditory processing deficits when induced at a neurodevelopmental point comparable to human “term,” but only transient deficits (undetectable in adulthood) when induced in a “preterm” window. Convergent evidence suggests that regardless of when or how disruption of early neural development occurs, the consequences may be particularly deleterious to rapid auditory processing (RAP) outcomes when they trigger developmental alterations that extend into subcortical structures (i.e., lower sensory processing stations). Collective findings hold implications for the study of behavioral outcomes following early brain injury as well as genetic/environmental disruption, and are relevant to our understanding of the neurologic risk factors underlying developmental language disability in human populations.

## Introduction

The profound plasticity of the developing brain affords an adaptive and often advantageous quality that is no longer prominent in adulthood (though recent research shows that the adult brain retains a greater level of plasticity than once thought). This early plasticity reflects the unique capacity of the developing brain to rapidly respond to external input and functional demands by enhancing, rerouting, or eliminating underlying neural circuitry—thus promulgating a brain (organism) more precisely suited to its unique environment. One important implication of this early and transient “responsiveness and optimization” capability is that *the developing brain is also potentially much less vulnerable to the detrimental effects of injury*. As the word “potentially” suggests, however, this principle is not straightforward. In order to tease apart the critical mechanisms and consequences of early brain disruption as indexed by later cognitive outcomes, it is quite valuable to employ animal models that allow us to map out the relative impact of clinically relevant neural manipulations (such as induced injury or genetic manipulations) on more basic outcome measures (such as rapid auditory processing (RAP)). Initially, in order to fully understand how the plasticity of early systems might contribute to an enhanced capacity to respond in a beneficial way to injury and/or disruption, it is important to briefly review key neurodevelopmental events for the central nervous system (CNS) in general, and the central auditory system in particular.

### A brief overview of CNS development in mammals

**Figure 1:**
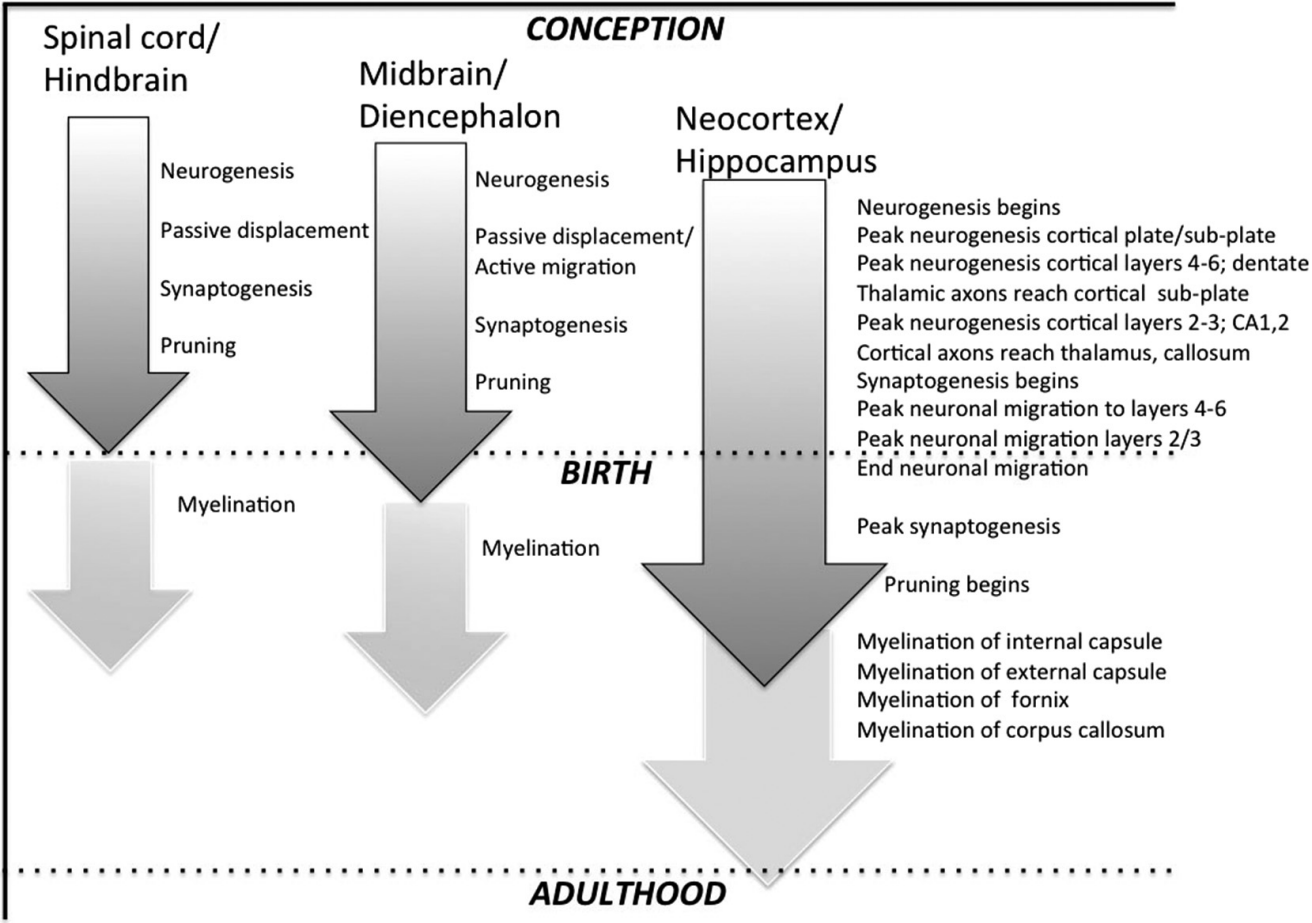
**Highly stylized depiction of regional neurodevelopmental stages in rat CNS.** Grey shaded arrows represent neuronal events starting with initial cell proliferation (neurogenesis), followed by migration (which occurs passively in many sub-cortical structures but actively in cerebral cortex), synaptogenesis, and pruning. Off-white arrows depict myelination, which largely begins postnatally, and continues into adulthood in many higher-order structures.

During embryonic development, the CNS arises from a specialized subset of epithelial cells (the neural plate). As the neural plate expands, the lateral edges fold in and merge, separating from the rest of the epithelium to create the neural tube (Nowakowski and Hayes, 2002; Diaz and Gleeson, 2009). In humans, formation of the neural tube occurs around embryonic day 26–28, and in rodents, around embryonic day 10.5 (E10.5). Following closure of the neural tube, regional specification begins, with the emergence of forebrain (rostral), midbrain, hindbrain, and spinal cord. In general, development proceeds along a “bottom-up” (or lowest-to-highest) gradient, with the spinal cord and hindbrain (caudal) structures maturing first. Around the end of the first gestational month in humans (E10–12 in rodents), proliferation of neural stem cells (fated to become neuroglia or neurons) begins, with the timing of local neurogenesis temporally staggered (again proceeding caudal to rostral or “bottom-up”; Rice and Barone, 2000; Nowakowski and Hayes, 2002; see Figure 1). Within the rostral-most forebrain, a highly proliferative area (destined to become neocortex) emerges along the surface of the lateral ventricles—the ventricular zone.

During the early stages of cortical cell proliferation, progenitors in the ventricular zone undergo symmetrical self-renewing cell division that generates additional progenitors. During this phase, some progenitors differentiate into radial glial cells (characterized by a long radial process that extends from the ventricular surface to the pial surface). During subsequent phases of neural proliferation, some radial glial cells continue to divide symmetrically in the proliferating zones of the cortex, but some radial glial cells undergo asymmetric cell division that results in the creation of one clone radial glial cell and a committed neural cell (post-mitotic neocortical neuron). The process of asymmetric cell division at this stage is known as neurogenesis. As post-mitotic neocortical neurons are born, they begin to migrate radially toward the pial surface, following the scaffolding created by radial glia (Nowakowski and Hayes, 2002; Diaz and Gleeson, 2009; Rakic, 2009). This process is called neuronal migration, and occurs between 13–21 weeks gestation in humans (Chong et al., 1996), and approximately embryonic day 14 (E14) to postnatal day 3 (P3) in rats. As an aside, it is important to note that in many lower areas of the CNS (spinal cord, brainstem) newborn neurons are moved into final laminar patterns through a passive displacement rather than active migration ( Figure 1).

During the initial stages of neuronal migration, the first post-mitotic neurons born in the ventricular zone migrate a short distance to form the cortical pre-plate. As new neurons are generated they continue to accumulate in the pre-plate, ultimately forming the cortical plate—which will in turn give rise to neocortical layers II–VI. The emergence of the cortical plate splits the pre-plate into the superficial marginal zone (layer I in the mature cortex) and the sub-plate below. Thus at this stage, the cerebral wall is characterized by four layers, including (from the most interior to most superficial): (1) the ventricular/sub-ventricular zone; (2) the intermediate zone; (3) the cortical plate/sub-plate; and (4) the marginal zone. During the next phase of neuronal migration, the cortical plate gradually develops more defined layers. Waves of newly generated neurons continue to migrate from the ventricular zone, past the sub-plate and into peripheral regions of the cortical plate, stopping short of the marginal zone. As a result, early born neurons are found in the deeper layers of the neocortex (layers V–VI), while later born neurons migrate beyond earlier migrating neurons to form the more superficial layers of the cortex (layers II–IV). This produces an inside-out pattern of lamination of the six cortical layers, as seen in both rats and humans (Rice and Barone, 2000; Nowakowski and Hayes, 2002; Diaz and Gleeson, 2009). Importantly, this process describes migration of excitatory glutamatergic pyramidal neurons (the bulk of cortical neurons), but neurons also migrate tangentially to reach their respective locations. Specifically, neurons that are destined to become inhibitory GABAergic cortical neurons show tangential migration, moving from their site of origin in the lateral and medial ganglionic eminence to their appropriate destinations in the cortex. This process typically is completed *after* radial migration ends, consistent with an initial hyper-excitability of immature cortex (i.e., tangential migration of inhibitory GABA neurons is delayed; see Marín and Rubenstein, 2001). Moreover, even as early GABAergic neurons complete migration and begin to form synaptic connections, they are initially excitatory (due to maturational shifts in intra-cellular/extra-cellular Cl^−^ gradients).

Once neurons settle into a permanent position in cortex, synaptogenesis begins. During this stage, neurons extend their axons (via dynamic growth cones) to locate a target region on another neuron. As with proliferation and neuronal migration, the window for peak synaptogenesis varies across the CNS, but again generally follows a “lowest to highest” (caudal to rostral) scheme. Notably, differences in the timing of synaptogenesis are seen even between cortical layers, yet the mechanisms remain largely the same (see Webb et al., 2001). In brief, growth cones on the leading edge of the growing axon contain receptors that detect local chemo-attractants, chemo-repellants, and cell adhesion molecules in the extracellular environment. The topographic pattern of these cues arises out of differential regional gene transcription and translation, leading to a complex extra-cellular pattern that “guides” axons to post-synaptic targets (e.g., see Rowitch and Kriegstein, 2010). Once a growth cone finds an appropriate postsynaptic target (soma or dendrite), the axon stops growing, and differentiates into a presynaptic terminal, while the target specializes into a postsynaptic site (Webb et al., 2001; Nowakowski and Hayes, 2002). Notably, although these early synapses are initially “functional,” they do not always function in the same manner as in adults (e.g., as indicated above, GABA is excitatory in early neurodevelopment but inhibitory in the mature brain; Ben-Ari, 2002). The first functional synapses emerge at approximately 27 weeks gestational age (GA) in human neocortex, with a peak in density around postnatal month fifteen (Huttenlocher and Dabholkar, 1997; Webb et al., 2001). In rats, the first functional cortical synapses are observed around E16, with peak synaptic density seen at approximately 3–4 postnatal weeks (P21–28; König et al., 1975).

Notably, at the same time that cortical neurons begin to seek intra-cortical targets (around P5 in rats), projecting axons from thalamic nuclei (whose terminals have been “waiting” in the sub-plate) begin moving into the cortex, seeking their target neurons in layer IV. The establishment of reciprocal cortico-thalamic and other subcortical projections proceeds slightly later, as cortical neurons in layers V and VI begin to extend *their* axons downward into the sub-plate, also seeking respective neural targets (Diaz and Gleeson, 2009). One interesting feature of this early process has particular relevance to the plasticity of young brains, and that is the fact that initial thalamo-cortical and reciprocal cortico-thalamic connectivity tends to be highly distributed and cross-modal (Katz and Shatz, 1996). This cross-modal connectivity in the very young brain is thought to give rise to unique re-organizational capabilities, such as the ability of temporal cortex to respond to visual stimuli in the congenitally deaf, and visual cortex to respond to somatosensory input in the congenitally blind (Bavelier and Neville, 2002). Based on these and other findings, it is believed that the immature brain can re-organize *across modalities* by retaining connections otherwise destined for pruning (Innocenti and Price, 2005). This process may also come into play in response to injury, for example as seen in the maintenance of ipsilateral motor connections that are retained when contra-lateral motor regions that would normally control function are injured (Johnston, 2009).

In general, it is believed that initial patterns of synapse formation in early development reflect a genetically mediated “best guess” of optimal neural configuration (Katz and Shatz, 1996), coupled with a dramatic exuberance in the production of neurons and synapses. As the brain matures, environmental stimulation (i.e., neural activation via input and action) facilitates the addition, elimination, and strengthening of synapses—allowing for further modification and refinement of neurocircuitry. Specifically (according to classic work by Hebb), synaptic circuits that receive the most activation persist and are stabilized, while circuits that receive little or no activation regress and are eliminated (Webb et al., 2001; Nowakowski and Hayes, 2002). This active elimination (pruning) of synaptic circuits continues well into postnatal life, with some areas of cortex (e.g., prefrontal cortex) pruning well into adulthood (early twenties in humans; Huttenlocher and Dabholkar, 1997). Myelination (or the formation of fatty sheaths around axons that increase speed and efficiency of conduction) also begins postnatally in rats and humans, and continues quite late in life. In fact, the proliferation of oligodendrocytes (pre-oligos) begins in the ventricular and subventricular zones largely *after* neuronal proliferation and migration is complete, and includes a “re-purposing” of radial glia (once their role in neuronal migration is over) into other forms of glia, including astrocytes (which support neurons) and oligodendrocytes (which produce myelin).

Behaviorally, the emergence of psychomotor and sensory functions necessary to perform more complex cognitive behaviors parallels the neurodevelopmental trajectory of structures and systems sub-serving those functions. That is, as different structures and neural systems come “on-line,” correlated behavioral capabilities simultaneously emerge (see discussion of these parallel trajectories in humans by Casey et al., 2005). As an example, P15 rat pups are unable to perform a rotarod task (a common behavioral tool to assess motor coordination), with adult-like patterns of performance on this task emerging around P20 (Bâ and Seri, 1995). Concurrently, underlying functionality of *cerebellum* and *basal ganglia* also approach an adult-like state around P21–28 (Bâ and Seri, 1995). Developmental changes in cognitive ability can also be observed as a function of structural maturation. For example, the hippocampus shows rapid maturation between P21–28 in the rodent (Bâ and Seri, 1995), and rodents also begin to show adult-like proficiency on spatial tasks such as the Morris water maze (a spatial learning and memory task) at this age (Bachevalier and Beauregard, 1993).

### Early development of the central auditory system

A bottom-up (lowest to highest) pattern of maturation is generally seen in the central auditory system, much as in other brain areas/systems. At the level of the inner ear, hair cells of the cochlea (which transduce sound waves into neural signals) undergo genesis and differentiation, and eventually form synapses with underlying spiral neurons of the auditory nerve. Once the cochlear apparatus and hair cells become functional, they are activated in response to stimulation of the tympanic membrane (or *in utero*, via bone conduction; Graven and Brown, 2008). In the immature system, spiral neuronal axons remain unmyelinated and of smaller diameter, accounting in part for initial long-latency responses to sound. The auditory nerve projects to the cochlear nucleus (CN), from which some ascending fibers cross to the contralateral superior olive (SO) and inferior colliculus (IC), and others synapse on the ipsilateral SO. The SO projects ipsilaterally to the IC, which projects to the medial geniculate nucleus (MGN) of the thalamus, and in turn to primary auditory cortex (AI). Development of functional connectivity between these structures appears to *precede* their peripheral activation by sound, with histologic evidence of synapses between hair cells, spiral ganglion neurons, CN and SO reported for rodents as early as E10–14 (Hoffpauir et al., 2009)—well before behavioral hearing onset (around P11–12, when the first indications of response to specific sounds are evident in rodents). Importantly, *spontaneous* activity—believed to be crucial to circuitry formation—can also be seen in these acoustic structures much earlier than P12 (i.e., before external activation, ascending propagation, and sound processing are evident; Tritsch and Bergles, 2010). Notably, core regions of these ascending structures are organized tonotopically (i.e., following an anatomic map characterized by progressive steps in the characteristic frequency producing maximal neural excitation, from low to high). This tonotopy is highly conserved in patterned ascending projecting systems in the adult brain, and is present in immature form (with initial representation mainly for mid-range frequencies) at the time of hearing onset (around P11 in rats; de Villers-Sidani et al., 2007).

In humans, the central auditory system reaches an initial milestone of maturity prenatally (onset of hearing), based on evidence of speech recognition for familiar voices in newborn infants, coupled with evidence of behavioral and auditory brainstem responses (ABRs) as early as 27–29 weeks of gestation (Sininger et al., 1997; Graven and Brown, 2008). However, the auditory system also continues to undergo considerable postnatal development, as evidenced by the high degree of behavioral plasticity, as well as changes in typical ABRs and auditory evoked potential response patterns (AERPs) across maturation. Indeed, while adult-like ABR to some low-frequency resolution tasks have been reported as early as 6 months (with high-frequency resolution developing slightly later in humans since higher frequencies are blocked *in utero*), many studies do not report adult-like ABR and/or AERP responses to more complex stimuli such as speech until much older ages (up to 16–18 years on some tasks; Fischer and Hartnegg, 2004).

Consistent with the generally later neurodevelopmental scheme in rats as compared to humans (with birth on P1 approximating mid-human gestation; Clancy et al., 2001; Workman et al., 2013), hearing and associated detectable ABRs do not come online in the rat until P11–12, with adult-like patterns of ABR and AERP emerging around P22 (depending on stimuli used). And as in humans, the ongoing development of higher acoustic structures undergoes substantial postnatal maturation. Over the period from P11 (approximate hearing onset) to P14, which has been identified as a “critical period” for plasticity in response to sound exposure in rats, de Villers-Sidani et al. (2007) report substantial expansion of the A1 cortical field, extension of high and low frequency representation, and decreases in neural thresholds and latencies to respond to sound. Moreover, tonotopic representation and response field properties are *highly* affected by experience during this window. For example, exposure of rats to chronic white noise during the first month of life results in deteriorated tonotopy, broader tone frequency tuning and degraded cortical temporal processing (as shown by poor response to rapid tone trains; Zhang et al., 2002; Zhou and Merzenich, 2008, 2009). Moreover, early exposure to noise appears to extend the “critical window” for auditory development, effectively prolonging immaturity of the system (Chang and Merzenich, 2003). Conversely, enriched postnatal exposure to tonal stimuli can enhance developmental precision and behavioral discrimination of sounds, with beneficial effects seen in rats following post-weaning acoustic enrichment and musical exposure (Engineer et al., 2004; Xu et al., 2009). Similarly, exposure to pulsed tones during development in rats broadens tone frequency tuning and results in an expansion of the A1 representations of the familiar tone frequency. Interestingly, these latter effects appear to trigger an earlier closure of the “critical window” (Zhang et al., 2001; de Villers-Sidani et al., 2007).

These developmental features characterize typical development of the central auditory system, but may also come into play in the neural response to early CNS disruption—particularly, disruptions known to alter auditory processing outcomes later in life.

### A brief historic overview on timing of brain injury and outcomes

Studies of the long-term behavioral consequences of brain injury as a function of age began in earnest with the extensive and seminal work of Margaret Kennard, who sought to assess the impact of early lesions in non-human primates on motor outcomes as a function of lesion timing, laterality, extent, and location. Although Kennard’s research did support a view that earlier lesions were at times less deleterious than comparable lesions at later ages, she also demonstrated that early lesions tended to lead to more negative outcomes when they occurred in regions that were closer to “functional maturity” at the time of injury (more like adult brains) as compared to more immature (later developing) regions. She also reported that initial evidence of sparing of function following early lesions could give way to emergent deficits later in life, with injured subjects failing to maintain typical maturation trajectories (reviewed in Dennis, 2010). Kennard supplemented these findings with her investigations of brain lesions and cerebral palsy in children, concluding that in general, the young brain had a remarkable capacity for re-organization following injury. Regrettably, interpretations of her research became over-simplified after her death, leading to the promulgation of the “Kennard Principle” (which Kennard never directly espoused). This view professed the simplified idea that behavioral recovery from brain injury would *always* benefit by occurring at an earlier time-point (i.e., the earlier an injury, the less severe the impact). This view was not entirely supported by Kennard’s own work, nor by subsequent research. For example, subsequent work has demonstrated that early lesions to *sub-cortical* areas can produce devastating behavioral consequences (e.g., Schneider, 1979). Thus early central auditory system disruptions that extend into sub-cortical structures (e.g., MGN, IC, CN) might exert more profoundly deleterious effects on long term acoustic outcomes as compared to higher order (cortical) disruptions.

More recently, Kolb and colleagues conducted a series of lesion studies on juvenile rats, assessing relative behavioral outcomes using both motor and learning tasks, as well as histologic measures taken *post mortem* (Kolb et al., 1983, 1984; Kolb, 1987; Kolb and Elliot, 1987; Kolb and Tomie, 1988). Results showed intriguing differences as a function of the timing of injury, as well as the effects of unilateral versus bilateral injury. Specifically, these researchers found that bilateral focal cortical lesions on P1 or P5 led to worse outcomes than those seen for adult rats with similar lesions. Interestingly, bilateral focal lesions on P10 led to greater sparing and improved performance relative to P1, P5, or adult lesions. Conversely, the effects of complete unilateral cortical ablation were relatively mild when performed < P14, with outcomes far better than were seen for adult rats with comparable ablation. Results clearly seemed to suggest that recovery from unilateral injury—even that of a dramatic nature (e.g., hemi-decortication)—is better in developing animals as compared to disruption in which homologous regions of both hemispheres are injured (Kolb, 1995). This intriguing principle could have important significance for the study of language disabilities, wherein researchers have long been puzzled by the fact that massive unilateral temporal lesions in early years still allow for language recovery, while individuals with no discernable neuropathology (at least as identifiable by current neuroimaging technology) can nonetheless exhibit profound language deficits (e.g., in specific language impairment (SLI) and/or dyslexia). This paradox suggests that developmental disruptions that occur bilaterally and very in early development (e.g., whole brain genetic or other prenatal risk factors) may lead to profound but subtle alterations in neural circuitry that are difficult to characterize via current technology, and yet could underlie robust changes in behavioral performance.

Additional research studies focused on the impact of early lesion timing (as measured by cognitive outcomes) have been conducted by Stiles and colleagues. These researchers assessed cognitive outcomes in language and visuo-spatial domains among infants and children with focal lesions (reviewed in Stiles et al., 2002). Results showed that: (1) patterns of long-term deficits depend greatly on when childhood lesions are incurred; (2) although early lesions do tend to lead to less pronounced deficits as compared to comparable lesions occurring later, subtle deficits can still be evidenced when the correct tasks are used; and (3) the pattern of outcomes differ (at least in humans) depending on whether lesions occur in the left versus right hemisphere, with children incurring early left lesions showing evidence of greater language preservation and recovery, but children incurring right lesions more likely to show persistent visuo-spatial deficits more comparable (though not as severe) as effects seen in similarly injured adults. The authors suggest that these disparities could reflect unique aspects of language organization in cortex, such as theories that language is protected at the expense of other domains in the developmental re-organization process (i.e., “crowding effects”). Alternately, it has been suggested that right hemisphere functions may be phylogenetically “older” and therefore more hard-wired (i.e., more difficult to shift to other uninjured cortical sites). Additional interpretations include the possibility that language functions show resilience to injury because of their more distributed nature, or that the relative timing of neural circuitry underlying language versus visuo-spatial functions may be “protective” to language. Overall, these findings have important implications for the study of outcomes in auditory processing following early brain injury, since aspects of auditory processing believed critical to language development (i.e., processing of rapid acoustic signals embedded in spoken language) may be more left-lateralized, while other aspects of auditory processing (e.g., processing of spectral components and music) could be preferentially sub-served by the right hemisphere (Okamoto et al., 2009)—at least in humans. The implications of such findings to small animal models where functional cortical lateralization is less evident remain unclear.

In summary, it is apparent that although long term outcomes following early brain disruption tend to be more adaptive following early injuries, many factors temper this phenomenon, including whether an injury is cortical or sub-cortical, unilateral or bilateral, left or right, and/or whether the incidence of injury occurs in a region and during a period of key neurodevelopmental events (e.g., window of peak proliferation or neuronal migration). In the following section we move to a discussion of research addressing specific neurodevelopmental mechanisms that might contribute specifically to anomalies in auditory processing and subsequent language development, with an emphasis on the possible role of differential plasticity as a function of the timing of early neural disruption.

## Auditory processing deficits and language disability: human populations, auditory processing, and animal models

### A neural signature for developmental language disability?

Given the “exceptions” to robust recovery from early neurodevelopmental disruption discussed, it is perhaps not surprising that developmental disorders—including developmental language disabilities—do occur, and with notable frequency. However, any early neural “plasticity” in response to underlying causal factors (such as genetic factors or undiagnosed injury or toxins) underlying these very early neurodevelopmental disruptions could reflect alterations in fundamental circuitry that occurred very early and are now hard to detect (i.e., via neuroimaging methods). In fact, a consistent underlying “neuropathological profile” or signature accompanying developmental disabilities of language tends to be subtle at best, and certainly very hard to identify—even by looking for “common denominators” across neurologic profiles of varied populations with developmental language impairment. For example, individuals with SLI and/or dyslexia show relatively subtle anatomic brain changes that require a large sample size for detection (e.g., alterations in asymmetry of the planum temporale and callosal cross-sectional area; see discussion by Leonard et al., 2006; Richardson and Price, 2009, for review). Similarly, populations with early hypoxic-ischemic (HI) injuries resulting from prematurity also exhibit poor language outcomes (among other anomalous cognitive measures; see Section Timing of Early Injury and Auditory Processing Outcomes in Rodent Models for further discussion), yet remarkably “normal” neural profiles (although subtle findings, such as abnormal fractional anisotropy in white matter circuits, appear to correlate with language outcomes in the preterm population; see Feldman et al., 2012). These results have puzzled researchers aiming to define the neural profile underlying developmental language disability, and to define specific neural substrates that might be studied in animal models (where experimental variables can be more easily and precisely controlled and studied). The remaining sections of this review further address this issue of a “neural substrate” for language disability, and our efforts to examine the relative impact of timing of “disruption of brain development” on behavioral outcomes relevant to the language domain (specifically, RAP) using rodent models.

### Subcortical anomalies and rapid auditory processing deficits in language disabled human populations

In 1985, Galaburda and colleagues published a groundbreaking report of focal cortical anomalies found *post mortem* in the brains of four dyslexic individuals. Histologic characteristics of these malformations strongly suggested a genesis in prenatal development, since they revealed abnormal placement of neurons within cortical layers (i.e., malformations including ectopias and microgyria). More recently, similar findings have been reported for individuals with developmental language impairment (Oliveira et al., 2007; Brandão-Almeida et al., 2008; Boscariol et al., 2010, 2011). Initially, these findings were thought to implicate a relationship between clinical diagnosis and specific disruption of fronto-temporal regions critical to language processing (since the distribution of anomalies in the affected brains was substantially greater in left perisylvian areas). However, subsequent studies demonstrated additional—lower level—anomalies in the same brains. Specifically, cellular anomalies in the lateral geniculate thalamic nucleus (LGN) and MGN were reported, with an excess of small neurons and a paucity of larger neurons in the thalamic nuclei of the dyslexic brains (Livingstone et al., 1991; Galaburda et al., 1994). In the LGN, this effect was attributed to disruptions specifically to the magnocellular sub-division, although in MGN, similar functional/structural sub-divisions have not been clearly identified (but see Stein, 2001). Moreover, related studies indicate that the reduction in large magnocellular cells of the LGN in dyslexic brains was likely associated with concurrent functional evidence that dyslexic subjects exhibit deficits in processing temporally relevant (magnocellular) aspects of visual information (i.e., low-contrast motion; Lovegrove et al., 1990; Livingstone et al., 1991; Slaghuis et al., 1992; Lehmkuhle et al., 1993).

Evidence of thalamic disruption in dyslexic brains led to a novel conjecture about the relationship between neuropathology and dyslexia. Specifically, the findings suggested that early disruption of developing cortico-thalamic projections could exert a cascading deleterious impact on *lower-level sensory processing*, and thus disrupt initial language development, and/or subsequent online processing (in both cases, a “bottom-up” phenomenon). In fact, recent and intriguing new research has shown processing anomalies at the level of the MGN (auditory thalamus) using neuroimaging technology in adult dyslexics during a phonemic processing task (Díaz et al., 2012). In accord with these findings, evidence of a concurrent reduction in large cells of the MGN of dyslexic brains (Galaburda et al., 1994) has been suggested to relate to consistent and wide-spread evidence that developmentally language disabled populations (including dyslexics) show deficits in processing rapidly changing aspects of auditory information. In fact, an early seminal series of studies by Tallal and colleagues showed that children diagnosed with SLIs were significantly worse than controls in discriminating fast (but not slow) tone sequences, and also were significantly worse than controls in discriminating consonant-vowel syllables with short, rapidly changing formant transitions (e.g., /ba/, /da/, /pa/, /ta/; see Tallal and Piercy, 1973a,b, 1975; Tallal, 1980, 2004; Tallal and Newcombe, 1978; Tallal and Stark, 1981; reviewed in Fitch and Tallal, 2003). Ongoing behavioral and psychophysical studies continue to accumulate demonstrating core deficits in RAP in varied developmentally language-disabled populations (McCrosky and Kidder, 1980; Reed, 1989; Robin et al., 1989; Watson, 1992; Neville et al., 1993; Farmer and Klein, 1995; Hari and Kiesla, 1996; Kraus et al., 1996; McAnally and Stein, 1996, 1997; Wright et al., 1997; Witton et al., 1998; Sutter et al., 2000; Renvall and Hari, 2002; Edwards et al., 2004; Cardy et al., 2005; Corbera et al., 2006; Au and Lovegrove, 2007; Cohen-Mimran and Sapir, 2007; Gaab et al., 2007; King et al., 2007).

Notably, although some critics suggest that auditory deficits could be simply co-morbid (parallel but non-causal) to language deficits (McArthur and Bishop, 2001; Rosen and Manganari, 2001; Ramus, 2003), ongoing research has revealed compelling evidence of robust longitudinal prediction. For example, Benasich et al. (2002, 2006) found that infants with a family history of language impairment or dyslexia (i.e., at an elevated risk of developing language problems; Tallal, 1980) were impaired relative to controls in the ability to discriminate two-tone sequences incorporating a short inter-tone interval, but not a longer interval. Longitudinal follow-up of these children revealed a strong relationship between early auditory processing thresholds and language outcomes at 12–24 months in both at-risk and typical groups. More recently, a similar relationship was seen for early AERP/EEG scores using the same two-tone sequences and language outcomes (Choudhury et al., 2007; Choudhury and Benasich, 2011). Predictive associations between early auditory processing skills have also been related to language performance in typically developing samples. Trehub and Henderson (1996) found that children who had performed above the median on a variety of acoustic gap detection tasks at 6 or 12 months were found to have larger productive vocabularies, use longer, more complex sentences, and produce more irregular words compared with children who had scored below the median. Such findings are supported by evidence from studies recording event related potential (ERPs) to auditory stimuli in infancy. Molfese and Molfese (1997) found that ERPs to consonant-vowel syllables recorded from infants within 36 hours of birth differed between children whose verbal IQ was above the norm at 5 years. Similarly, infants with a family history of dyslexia showed different patterns of ERPs to consonant-vowel stimuli as compared to matched controls at 1 week and at 6 months (Leppänen and Lyytinen, 1997; Leppänen et al., 1999; Pihko et al., 1999; see also Leppänen et al., 2012).

Collectively, the data clearly support the notion that the ability to make fine grained auditory discriminations (RAP) is strongly related to later language development, and that deficits in this basic function may impair subsequent language development—with ultimate implications for higher-order processes (such as reading) that are seemingly distal to (i.e., far downstream/upstream from) basic acoustic processing. These and other findings argue convincingly for a relationship between early acoustic processing capabilities (such as might be affected by disruption to auditory thalamic structures such as the MGN), and long-term language outcomes. Based on these links, a theoretical “next step” was to examine the neurodevelopmental underpinnings for this functional language “pre-cursor”—RAP—in a non-human model.

### Animal models of rapid auditory processing deficits

**Figure 2:**
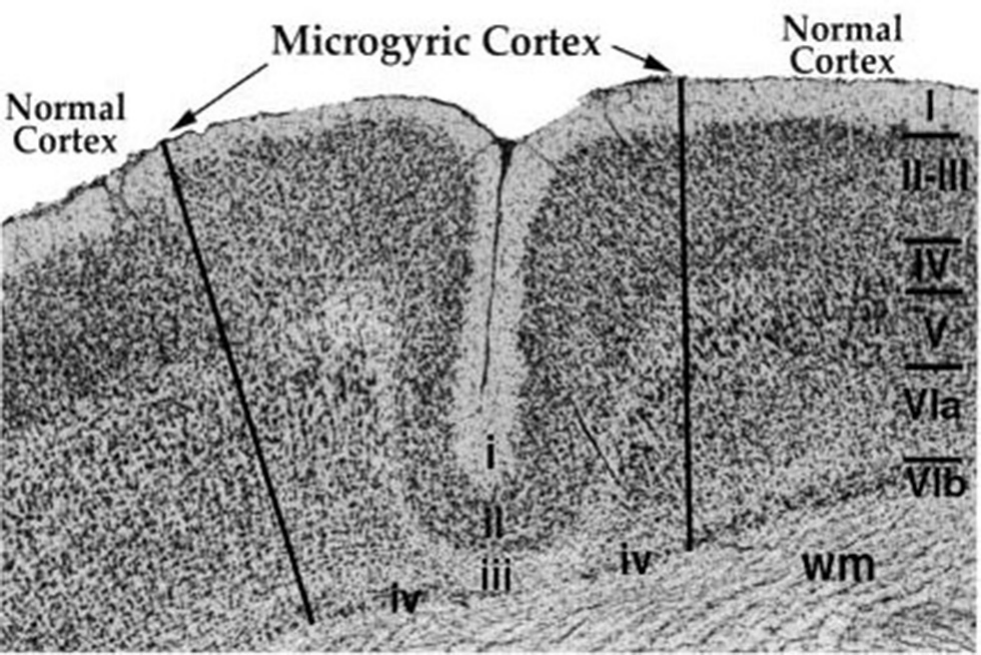
**Sample microgyric lesion in rat parietal cortex following a P1 focal freezing lesion.** The six normal cortical layers are denoted with the Roman Numerals I–VIb, and wm denotes cortical white matter. The microgyric cortex is composed of cortical layers i–iv and shows a distinct fold in otherwise smooth cortex. From Peiffer, A.M., Rosen, G.D. and Fitch, R.H. (2002). Rapid auditory processing and MGN morphology in rats reared in varied acoustic environments. *Dev. Brain Res.* 138, 187–193.

Initial efforts in developing an animal model for RAP deficits focused on evidence that induction of a focal freeze lesion to cortex of a 1-day-old rat pup (performed through the skull cap, which is very thin at this age) would lead to the subsequent formation of a microgyrus—a focal region of cortex characterized by anomalous cortical layers (thus indicative of abnormalities in migration; see Figure 2). Microgyri induced in this manner were found to be remarkably histologically similar to the microgyria identified by Galaburda et al. (1985) in *postmortem* human dyslexic brains (Dvorák and Feit, 1977; Humphreys et al., 1991; Rosen et al., 1992). Subsequent research revealed that rats with induced unilateral or bilateral microgyria consistently evidenced deficits in RAP—deficits remarkably similar to those seen in children and adults with language dysfunction (note that auditory processing deficits were greater for rats with bilateral microgyria and/or bilateral double microgyria; Fitch et al., 1994; Clark et al., 2000a,b; Rosen and Manganari, 2001; Peiffer et al., 2002, 2004b; Threlkeld et al., 2009). Moreover, these same microgyric rats showed anatomic disruptions in the MGN, also similar to those seen in human dyslexic brains (i.e., a shift in cell size distribution toward smaller cells as compared to sham MGN; Herman et al., 1997; see also Peiffer et al., 2002). It remains unknown whether the shift in cell size in the MGN associated with these induced cortical anomalies reflects a loss of large MGN neurons, or some other developmental mechanism.

Importantly, the behavioral RAP deficits found in microgyric rats were seen concomitantly with normal performance (comparable to shams) on easier acoustic tasks that *did not incorporate a temporal demand*, such as simple tone detection, long silent gap detection, or discrimination of two-tone sequences with longer inter-stimulus intervals. Moreover, additional research revealed that microgyria-induced RAP deficits were particularly evident in juvenile rats, as compared to these same subjects when tested in adulthood (Friedman et al., 2004; Peiffer et al., 2004a). Specifically, whereas young microgyric rats exhibited RAP deficits that could be elicited on relatively simple (but still temporally demanding) tasks such as short gap detection, more complex rapid processing tasks (such as discrimination of two-tone sequences with short intra-stimulus intervals) have been used to elicit more robust deficits in older microgyric rats. These findings may parallel similar developmental trends seen in child versus adult human dyslexic populations—specifically, that impairments in silent gap detection thresholds are seen in dyslexic children, but are no longer seen in dyslexic adults (Hautus et al., 2003). Also, these findings may be consistent with suggestions that while some of the more basic sensory processing deficits in language disabled populations may remediate with age, the long-term consequences of those early deficits (as measured by language processing) may persist.

Given these multiple parallels between the emergence of RAP skills in an animal model, and human clinical data, we set out to explore more specifically the parameters governing the relationship between early brain disruption and auditory discrimination outcomes in a rodent model. This approach included a series of studies examining the relative impact on long-term processing of rapidly changing acoustic information following different *types* of early brain injury, as well as different *timing* of injuries, in efforts to provide insights about how and when the brain might respond to disruptions/injuries that are relevant—in human populations—to long-term language outcomes (for further discussion see Fitch et al., 1997a; Fitch and Tallal, 2003). Importantly, for all of the studies described below, easier versions of acoustic tasks were also used to ensure that impaired subjects could hear and process basic sounds. These distinctions are critical in pointing out that we are not modeling a generalized learning or sound processing disorder but rather, a deficit specific to the processing of rapidly changing (short duration) acoustic stimuli.

## Timing of early injury and auditory processing outcomes in rodent models

### A rat model of cortical neuronal migration anomalies and RAP outcomes

**Figure 3:**
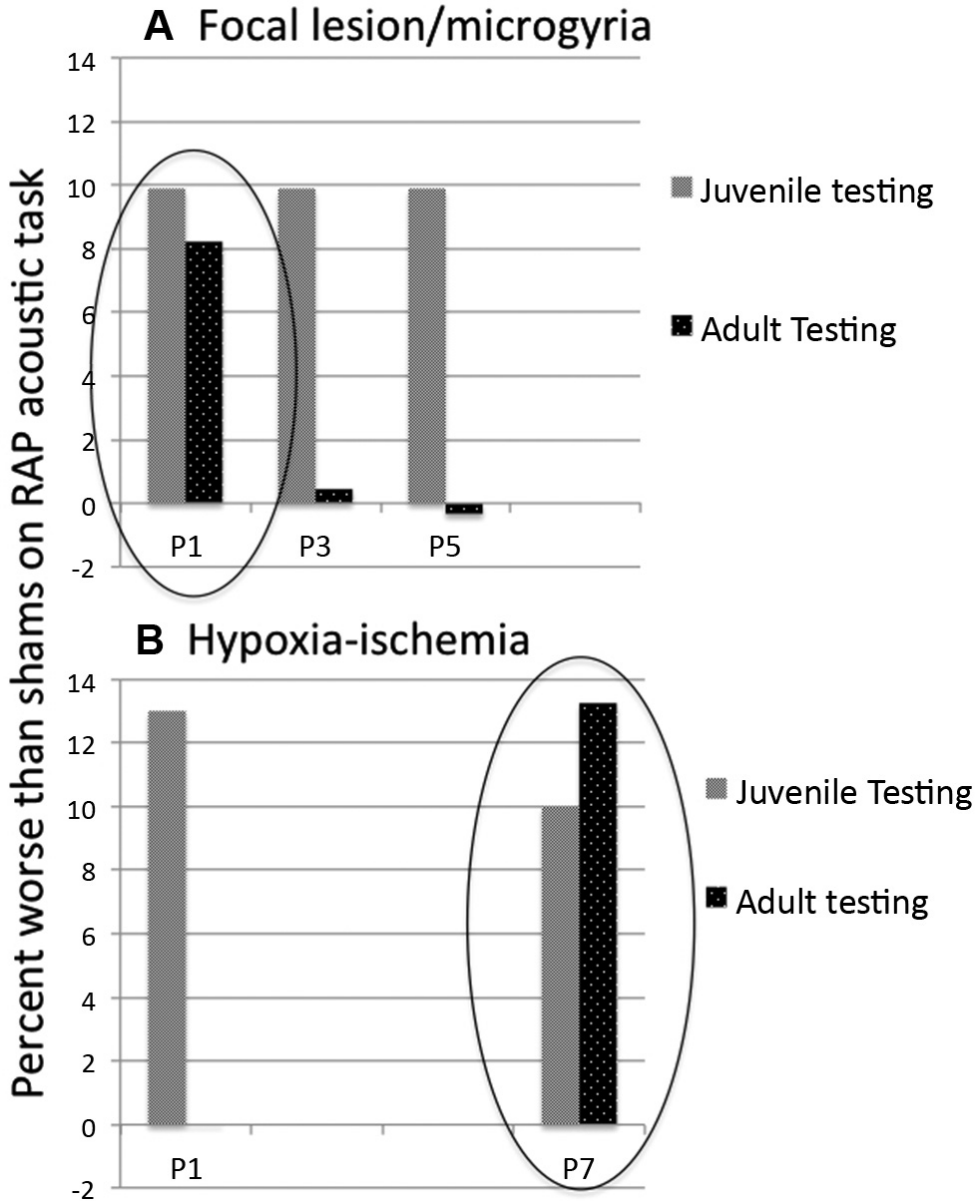
**Mean difference in percent attenuation as compared to matched shams on a rapid acoustic processing discrimination task.** Behavioral measures were obtained in juvenile (about P30) and adult (about P60) rats (repeated testing, same subjects). **(A)** Subjects with lesions induced on P1 and P3 showed cortical microgyric malformations in *post mortem* analysis. Subjects with P5 lesions showed only glial scarring. All subjects showed deficits relative to matched shams in juvenile testing. In adulthood, only P1 lesioned (microgyric) subjects were significantly impaired as compared to shams. Data adapted from Threlkeld et al. (2006). **(B)** For subjects with HI induced on P1 and P7, both groups again showed deficits in the juvenile period, but only P7 HI remained impaired relative to matched shams in adulthood. Data adapted from McClure et al. (2006).

To further examine the underlying neurodevelopmental events that may contribute to functional RAP deficits, we investigated silent gap detection capabilities in juvenile and adult rats that received bilateral freezing lesions or sham surgery on P1, 3 or 5 (Threlkeld et al., 2006), following on the procedure described earlier that leads to cortical microgyria when performed on P1 (as described in Section Animal Models of Rapid Auditory Processing Deficits, see Figure 2). The behavioral task was developed based on the widely held view that the ability to detect a very brief silent gap in a white noise background is a good measure of fine-grained temporal acoustic acuity, particularly at very short durations. As such, we employed gap durations between 0 and 10 msec (although easier/longer duration stimulus versions of the task were also used for comparison). This silent gap detection task was embedded in a pre-pulse inhibition paradigm (allowing us to assess rodent processing thresholds without a need for training and learning confounds; Fitch et al., 2008). The timing of the lesions on P1, P3 and P5 was selected based on evidence that, relative to human neurodevelopmental milestones, these dates would correspond roughly to human GA’s 20, 25, and 30 weeks (i.e., prenatal development; Clancy et al., 2001; Workman et al., 2013). Importantly, the critical neurodevelopmental events ongoing in the rat brain during this period include the end of neuronal migration to upper cortical layers—which is largely completed by P2–3 in rats (although cortical neuronal migration is entirely prenatal in humans). Consistent with this timeline, our histology revealed classic “microgyria” in P1 and 3 focal lesioned rats, but *not* in the P5 lesion group (which only showed evidence of glial cortical scarring). We also found a significant reduction in brain weight and neocortical volume in P1 and 3 lesioned (microgyric) brains relative to shams (Threlkeld et al., 2006), as well as graded reduction in the size of the corpus callosum that was most evident in P1 lesioned (microgyric) subjects (Threlkeld et al., 2007b). In terms of behavioral outcomes, RAP scores (on the 0–10 msec silent gap task) from subjects in the juvenile period revealed significant RAP deficits in *all three lesion groups* as compared to sham subjects, but adult (P60+) data revealed a persistent disparity *only* *between P1-lesioned (microgyric) rats and shams* (Threlkeld et al., 2006; Figure 3A).

Importantly, we have reported previously that the cortical location of lesion/microgyria induction is not a critical variable in eliciting later RAP deficits in rats (Herman et al., 1997). That is, focal bilateral lesions induced in *parietal, visual, or pre-frontal* cortex were all found to lead to RAP deficits in rats (as measured on silent gap detection tasks), regardless of lesion location. In fact, the standard microgyria induction protocol for the P1/3/5 timing study described above used lesion induction directed at *parietal* and not temporal cortex (Threlkeld et al., 2006). Thus convergent data suggest that some form of *generalized* pathology affecting overall neocortical and/or cortical/sub-cortical development is responsible for these emergent RAP deficits, rather than factors specific to the local formation of microgyria in auditory cortical areas *per se*. This is also consistent with evidence (described above) that the window for the induction of RAP deficits via focal disruption of cortical neuronal migration is constrained to *the window during which neuronal migration occurs* (i.e., < P3 in rats). We hypothesize that a disruption to the formation of cortical layers in a focal region of cortex (through ischemic necrotic death of middle cortical layers) may initiate a cascade of developmental changes impacting on cortico-thalamic connectivity—leading in turn to developmental changes in the thalamus itself. This latter view is consistent with evidence that cellular changes in the MGN induced by microgyria formation are *also* seen regardless of microgyria location in cortex (Herman et al., 1997). Interestingly, even though the development of cortico-thalamic projections is still ongoing in rats at P5 (Diaz and Gleeson, 2009), the fact that cortical layering is largely established at this time may minimize the subsequent disruption to the developmental cascade, with *transient* rather than permanent effects evident on functional auditory processing (RAP) in rats that received a focal lesion when cortical layers were largely in place (P3–5; Threlkeld et al., 2006; Figure 3A).

### A rat model of premature versus term hypoxic-ischemic (HI) injury and RAP outcomes

In addition to collective findings linking cortical neuronal migration anomalies with deleterious long-term language outcomes, other forms of early brain disruption are also associated with impaired long-term language outcomes. In particular, a major cause of brain injury among neonates involves HI injuries, reflecting compromised blood and/or oxygen delivery to the brain.

In premature/very low birthweight (VLBW) infants, brain injury can arise due to fragile cerebral vascular systems as well as poor auto-regulation. Specifically, blood pressure fluctuations can lead to ruptures, which in turn can result in intraventricular hemorrhage (IVH; bleeding within the ventricles) or periventricular hemorrhage (PVH; bleeding surrounding the ventricles; Volpe, 1997, 2009). Ischemic re-perfusion failure, characterized by collapse of capillaries during low blood pressure fluctuations followed by failure to re-perfuse, can also lead to non-hemorrhagic HI injury (e.g., periventricular leukomalacia PVL; Volpe, 2001). PVL is associated with a loss of white matter surrounding the ventricles. Similarly, HI injuries can arise in term infants, typically following birth complications such as cord prolapse, placental disruptions/failure, and/or cord asphyxia (Johnston et al., 2001; Volpe, 2001; de Vries and Cowan, 2009; Lai and Yang, 2011). Due to the more global nature of these insults, full term infants with HI events are more commonly diagnosed with hypoxic ischemic encephalopathy (HIE), and show damage in predominantly gray matter areas such as cortex, hippocampus, basal ganglia, and thalamus (Huang and Castillo, 2008; Martinez-Biarge et al., 2011).

Not surprisingly, both preterm and term HI populations exhibit long-term disruptions in language abilities. For example, children born very prematurely are at elevated risk for early language delays (Foster-Cohen et al., 2007), and show deficits in spelling, reading, and writing, as well as receptive and expressive language (Ortiz-Mantilla et al., 2008; Luu et al., 2009; Van Lierde et al., 2009). Early language measures also predict later language scores in this population, for example with comprehension scores at 4 years correlating with later performance on language comprehension, naming, and auditory discrimination tasks (Jansson-Verkasalo et al., 2004). At age 6, these same subjects showed alterations on mismatched negativity during naming tasks and difficulty in pre-attentively discriminating changes in syllables (Jansson-Verkasalo et al., 2004). Full term infants with moderate to severe HIE also show receptive language, reading and spelling scores in childhood that are significantly lower than healthy full term control scores (Badawi et al., 2001), and correlations can be found between verbal IQ and degree of injury (Steinman et al., 2009). Importantly, researchers have also demonstrated that children diagnosed with severe PVL lesions at birth show deficits on RAP tasks later in childhood (Downie et al., 2002), opening the door to behavioral assessments of RAP in animal models of induced neonatal HI injury as a possible window to neuropathological underpinnings of language difficulties in this population.

**Figure 4:**
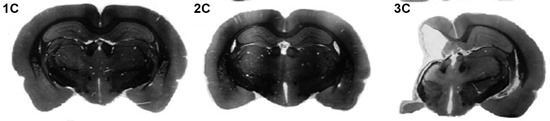
**Coronal cross-sections (nissl) from exemplar adult rats following sham (1C), P1 HI (2C) or P7 HI (3C) injuries.** Taken from McClure, M., Threlkeld, S., Rosen, G. and Fitch, R.H. (2006). Rapid auditory processing and learning deficits in rats with P1 versus P7 neonatal hypoxic-ischemic injury. *Behav. Brain Res.* 172, 114–121.

Fortunately, animal models can provide further insight into the neuroanatomical and behavioral features of neonatal HI injury, for example using the Rice-Vannucci method (Vannucci and Vannucci, 2005). This model entails cauterization of the right common carotid artery followed by exposure to a less than normal oxygen environment for a period of time (typically 8% oxygen (as opposed to the normal 21% partial pressure) for 90–150 min; Vannucci and Vannucci, 2005). Induction of HI injury using this method in rodents between P1–5 can produce injuries that correspond roughly to those seen in premature/VLBW infants with HI, including ventriculomegaly and predominantly white matter damage (much like human PVL; Scafidi et al., 2009; Figure 4). Conversely, injury induced between P7–10 leads to neural anomalies that appear to correspond to term birth HI injury, with gray matter damage predominating (as in the case of HIE; Vannucci and Vannucci, 2005; Figure 4). These differential neuropathological profiles open the door to experimental assessment of the impact of timing of induced HI on neuropathogy and associated long-term RAP profiles.

Recently, we performed a study to characterize the similarities/differences in RAP and other behavioral outcomes following early (P1–3) and late (P7) HI injury in rats. Male rats with comparable HI (same period of hypoxia) but induced on P1/P3 *or* P7, as well as sham controls, were tested on a variety of behavioral tasks in both juvenile and adult periods. Results showed that all groups could hear normally, and could comparably perform simple sound processing tasks (e.g., single tone detection and long-gap detection). However, P1/P3 HI animals showed only *transient* deficits on RAP tasks (in the juvenile period but not in adulthood) as compared to shams (McClure et al., 2006; Alexander et al., submitted-1; Figure 3B). P7 HI animals, conversely, exhibited *persistent* deficits in processing rapid acoustic information across both juvenile and adult periods (see Figure 3B). Also, P1–3 HI animals did not show any significant reductions in brain volume that we could detect, although substantial reductions in the volume of right cerebral cortex, hippocampus and striatum (as measured by stereologic reconstruction) were seen in P7 HI rats. Interestingly, P7 HI rats also showed a significant shift to more small and fewer large neurons in the MGN, an effect that was *not* seen in P3 HI subjects (Alexander et al., submitted-2).

Here, our results appear to contradict the findings of Threlkeld et al. (2006), where we found that a focal induced ischemic lesion leading to the formation of microgyria had the most significant effects on RAP, brain weight, and callosal area when performed on P1, rather than P3 or P5 ( Figure 3A). Using an induced HI injury, we found virtually an opposite effect—that subjects with P1/3 HI had only transient RAP deficits, while those with P7 HI had *permanent* robust RAP deficits as well as significant loss of neural tissue in a variety of regions, and a shift in MGN cell size towards more small and fewer large neurons (an effect also seen in microgyric rats when lesions were induced on P1; Herman et al., 1997; Figure 3B). The possible implications of these combined findings are discussed further below.

## Auditory experience and amelioration of auditory deficits in rodent models

A key final note is that we have found an important role for age of testing in eliciting RAP deficits associated with early neural disruption, and we have also found an impact of prior experience on outcomes during later testing. Specifically, we performed a study in which male rats received bilateral induced microgyria (via focal ischemic cortical lesions on P1, see Section Animal Models of Rapid Auditory Processing Deficits), while comparable sham littermates were retained as baseline controls. In addition, a subset of these animals were tested on auditory processing tasks as juveniles, while their counterparts remained undisturbed until adult testing, when all animals again received a full battery of auditory discrimination assessments. Results were extremely intriguing. First, test results from naïve juvenile rats compared to naïve adult rats showed a small maturational improvement in auditory processing acuity (with better performance and lower thresholds in adults). Second, results showed that the performance of adult shams that *received juvenile testing* improved orders of magnitude more than was seen from endogenous (undisturbed) maturation alone. Third, we found that the microgyria-associated deficits in RAP, which were significant in our juvenile samples, were *no longer seen* when these same rats were tested as experienced adults. However, when examining the naïve adult cohort, significant deficits among the microgyric subjects on RAP tasks *were* found (Threlkeld et al., 2009). These results point to critical issues regarding the role of assessment in defining disorders—specifically where prior experience has occurred, such that underlying deficits may be masked or even remediated. Normal human development entails substantial experience of varied and complex nature, and thus our ability to assess and define critical underlying processing deficits in older populations (as is necessary to disentangle the neurologic and behavioral underpinning of higher-order dysfunction) is called into question. In fact, the results described above may help to explain why evidence of basic deficits can be subtle or may even fail to be replicated across studies with clinical language disabled populations. On the other hand, the ability to test infants and small children is constrained by our inability to diagnose language difficulty until relatively late milestones fail to be achieved. This conundrum represents a huge issue in human clinical language disability research, and clearly highlights one reason that animal research is crucial to a complete understanding of the mechanisms at play in the complex process of emergent developmental disorders of language.

## Discussion

The research reviewed here highlights several principles of developmental response to brain injury and the role of timing. First, it is clear from a vast literature that the young brain is indeed “plastic,” and in many cases can respond more effectively to external input (as measured by learning) when compared to the adult brain. Moreover, these adaptive features of plasticity can extend to the response of the developing brain to disruption, where positive compensatory and/or adaptive responses to injury (leading to functional optimization) are often seen in the young brain. However, this latter extension of the beneficial effects of “early plasticity” must be qualified. Specifically, the parameters determining whether the developmental response to a disruption (injury, mutation, toxin) will be “adaptive” or “maladaptive”—or what Giza and Prins (2006) call “good” versus “bad” plasticity—remains something of a mystery. Indeed, it is difficult to ascertain how brain mechanisms *can* be uniquely responsive to immediate cues in deploying patterns of reorganization that will provide an optimal compensatory outcome down the road (as opposed to an *even more deleterious* behavioral outcome) following a given disruption. And the answer may be that the brain is only *co-opting* existing mechanisms that evolved to support maximal early development and learning. Indeed, it seems unlikely that evolutionary pressures acted directly on mechanisms of response to brain injury *per se*, since strong reproductive contributions after such injuries seem unlikely. Accordingly, in some cases, *patterns of re-organization and/or compensation to early disruption may actually be worse than if no re-organization had occurred at all* (Schneider, 1979; Giza and Prins, 2006).

### Windows of vulnerability

Certainly, experiencing injury or disruption during a window of heightened vulnerability (i.e., a period of critical processes) can be one impediment to optimal reorganization. In this case, disruption of a key process that cannot be reproduced or mended by following an “alternate route” appears to cause permanent deleterious consequences. Periods of peak neurogenesis, for example, represent windows of particular vulnerability for fetal exposure to radiation and toxins (Rice and Barone, 2000). Similarly, cortical disruption during peak periods of migration, or during later periods of neuronal maturation and critical synaptogenesis, may lead to long term deficits that might not be seen in response to the same disruption at a slightly earlier or later point. We suggest that this interpretation may explain why focal freezing lesions to the cortical plate that produce microgyria lead to lasting RAP deficits when induced on P1, but not on P5 after neuronal migration is completed (Threlkeld et al., 2006). These cortical anomalies may in turn lead to deleterious developmental changes that ultimately alter sub-cortical functions (e.g., MGN; Herman et al., 1997).

This interpretation is consistent with findings from other developmental manipulations we have performed that also produce RAP deficits in rodent models, for example the* in utero* knock-down of dyslexia risk genes. Specifically, evidence has identified both Kiaa0319 and Dyx1C1 as risk genes for dyslexia, and concurrent animal research shows that both genes are involved in regulating early neuronal cortical migration (E14—P3 in rats; Galaburda et al., 2006). Accordingly, the RNAi knock-down of the rodent homolog’s for these proteins (transfected into newborn ventricular zone neurons) would be expected to impair the cortical neuronal migration process—and in fact, migrational anomalies are seen in the cortex of both Kiaa0319 and Dyx1c1 RNAi rodent models (Galaburda et al., 2006). Importantly, we found also that these* in utero* manipulations led to later RAP deficits in these same rats (Threlkeld et al., 2007a; Szalkowski et al., 2012, 2013). Recently published related research has also demonstrated anomalies in neuronal encoding of speech stimuli in cortical neurons from rats transfected embryonically with Kiaa0319 (Centanni et al., 2013). And, in Dyx1c1 RNAi animals, a shift in cell size of the MGN (towards more small and fewer large cells) was also found (Szalkowski et al., 2013). Again, these findings point to the critical consequences of disrupting cortical neuronal migration.

### Bilateral versus unilateral injury

Another factor in interpreting the experimental data presented here is that induced cortical microgyria (though small and focal) were *bilateral* (see Section Animal Models of Rapid Auditory Processing Deficits for details), whereas our more severe HI injury was *unilateral* (noting that some injury to hemisphere contra-lateral to carotid ligation can occur, but most pathology measures fail to show significant cell death or tissue loss from the period of reduced oxygen alone). Thus our HI findings appear consistent with those of Kolb (1995), who showed that recovery from very early *bilateral* injuries is particularly poor, whereas rats showed remarkable preservation of cognitive skills following complete hemi-decortication during this same early window (Kolb, 1995). Indeed, the bilateral nature of our induced microgyria versus unilateral HI injury could account in part for different patterns of outcome on RAP tasks. In support of this view, a related study examined auditory outcomes as measured by A1 neuronal recordings in rats subjected to complete anoxia (0% oxygen) for about 15 min on P1 and again on P2. Here—unlike induced HI injuries that employ a coupling of unilateral carotid ligation and prolonged reduced oxygen to produce a unilateral injury—rats were subjected to a very severe anoxic incident impacting both hemispheres equivalently. Interestingly, the authors of this study found that after an anoxic incident on P1 and P2, acoustic responses from neurons in A1 as measured in adulthood (P90+) were significantly degraded (Strata et al., 2010). Changes included broader tuning curves, increased latencies, reduced response amplitudes, and a degraded capacity to follow high-rate repetitive stimuli. Authors suggest that although measures were recorded from the cortex, anomalies in processing may very well have arisen at lower levels of the auditory system (e.g., CN, IC, or medial geniculate; see also Strata et al., 2005), but anomalies did not appear to reflect direct damage to cochlear mechanisms (based on histology; Strata et al., 2010). Although Strata and colleagues did not perform behavioral assessments, these findings supplement our own in showing that a severe *bilateral* developmental disruption on P1 or P2 can produce lasting deficits in acoustic signal processing, even though a severe *unilateral* HI injury on P1 or P3 failed to exert permanent effects on RAP (McClure et al., 2006; Alexander et al., submitted-1).

### Cortical versus subcortical anomalies

Here we return again the evidence that—across various developmental rodent models we have successfully employed to elicit persistent behavioral RAP deficits (including P1 induced cortical microgyria, P7 HI, and *in utero* RNAi transfection with Dyx1c1)—animals demonstrating RAP deficits *consistently also* show significant cellular anomalies in the MGN. In the P1 microgyria model, these anomalies *must* be secondary to cortical disruption, since no direct injury was induced in thalamus. Similarly, in RNAi knock-down of Dyx1c1, MGN anomalies are seen along with migrational abnormalities in cortex (including ectopias, microgyria and band heterotopias) that reflect direct transfection of newborn cortical neurons in the ventricular zone. In the case of induced HI or anoxia, of course it is possible that damage to the MGN occurs through direct injury, but equally possible that the substantial injury to cortex exerts deleterious effects via corticothalamic developmental feedback. Overall, cumulative findings consistently point to the fact that, despite the relatively profound plasticity of the developing cortex (i.e., evidence that many early cortical injuries can be compensated through reorganization and/or other forms of plasticity), injuries that trigger developmental changes that *cascade into sub-cortical structures* may lead to profound and lasting deficits for which the developing system is unable to compensate. Such effects may be particularly profound when they occur in neural substrates upon which critical and distributed cognitive processes—such as language—are built. This assertion is consistent with evidence that subcortical indices of speech/language processing are highly predictive of higher order language difficulties in children (Hornickel et al., 2009; Díaz et al., 2012), that language impairments associated with sub-cortical anomalies tend to be more severe (Aram and Eisele, 1994), and also that volumetric measures in subcortical regions can accurately predict language outcomes (Ortiz-Mantilla et al., 2010).

## Conclusion

Cumulative evidence presented here suggests that developmental neuronal reorganization triggered by disruption—regardless of when (within the early postnatal window we examined) or how disruption occurs—that alter *subcortical* development in some way may have particularly maladaptive consequences for later ability to process rapidly changing acoustic information. This latter point may explain why profound impairments in critical processes such as language can be evidenced even when a brain appears to be anatomically “normal” at a gross level. In effect, developmental “rescue” mechanisms may have been deployed in response to whatever underlying deviations occurred (i.e., genetic, toxins, injury), yet these mechanisms failed to prevent a deleterious functional outcome. In fact, re-organizational mechanisms as implemented may have produced *worse* outcomes. Moreover, negative consequences may be particularly pronounced as measured by processes that are highly dependent on speed of processing (which requires optimal neural efficiency)—such as the discrimination of rapidly changing sensory input.

In closing, a review of our data—in combination with that of many others—supports the position that the developing brain responds very differently to injury as compared to the adult brain, and that in many cases this response is in fact adaptive. Indeed, infants and children show overall better cognitive outcomes following injuries and disruptions that would be devastating to an adult brain. On the other hand, some complex higher order processes—particularly the unique process of language (which requires optimal processing at *both* low levels of the auditory system (to encode speech), as well as optimal complex encoding at higher levels of cortical language-specific areas)—can be particularly vulnerable to developmental shifts that *alter critical sub-cortical processing stations*. And although RAP deficits may have minimal effect on species survival and evolutionary fitness in a non-lingual species such as rodents, humans—who have evolved complex higher order processes that are integral to the ability to function in society—show devastating behavioral impairments that include disruptions of critical language and reading development. Future research is needed to address how reorganizational mechanisms leading to alterations in subcortical morphology might be triggered, and how interventions might be employed to guide the developing CNS to an optimized neural “system” following disruption that would preserve RAP functions critical to later language development.

## Conflict of interest statement

The authors declare that the research was conducted in the absence of any commercial or financial relationships that could be construed as a potential conflict of interest.
